# Nano/microencapsulation of feed additives for ruminal microbiome modulation and enteric methane mitigation in ruminants: a critical review

**DOI:** 10.3389/fvets.2026.1798669

**Published:** 2026-05-05

**Authors:** Edwin Oswaldo Botia Carreño, Mona Mohamed Mohamed Yasseen Elghandour, Pasquale De Palo, Aristide Maggiolino, Maria De Angelis, Abdelfattah Zeidan Mohamed Salem

**Affiliations:** 1 Doctorado en Ciencias de la Producción y de la Salud Animal, Facultad de Medicina Veterinaria y Zootecnia, Universidad Nacional Autónoma de México (UNAM), Ciudad de México, Mexico; 2 Facultad de Medicina Veterinaria y Zootecnia, Universidad Autónoma del Estado de México, Toluca, Estado de México, Mexico; 3 Dipartimento di Medicina Veterinaria, Università degli Studi di Bari Aldo Moro, Valenzano, Italy; 4 Dipartimento di Scienze del Suolo, della Pianta e degli Alimenti (Di.S.S.P.A.), Università degli Studi di Bari Aldo Moro, Bari, Italy

**Keywords:** animal health, animal production, encapsulation, nanotechnology, probiotics, ruminal microbiota

## Abstract

The latest advances in nutrition, microbiology, and omics sciences are redefining strategies to improve health indices and productivity in livestock. A novel strategy focuses on the deliberate modulation of rumen and intestinal microbiome ecosystems, which, besides being complex, are crucial for animal performance. The use of feed additives, such as bioactive compounds derived from plants and probiotics, has a long tradition supported by their known antioxidant, anti-inflammatory, and antimicrobial properties, among others. However, their practical efficacy is often compromised by their high susceptibility to degradation. Environmental factors such as light, temperature, and harsh conditions like extreme pH and enzymatic and microbiota activity in the gastrointestinal tract of livestock can inactivate these compounds before they reach their site of action to exert the beneficial effects mentioned above. To solve this challenge, nanotechnology, specifically micro- and nanoencapsulation techniques, presents an innovative solution. These strategies can protect bioactive compounds, providing controlled release and targeted delivery to specific absorption sites. This design not only optimizes probiotic survival and bioavailability of bioactive compounds but also facilitates more effective modulation of ruminal and intestinal microbial communities. Recent evidence indicates that this modulation translates into tangible productive benefits, such as better nutrient absorption and higher energy efficiency, positively impacting parameters like milk production. Additionally, these encapsulation techniques improve the efficiency of these bioactive compounds to mitigate enteric methane emissions by altering ruminal fermentation patterns. This review critically analyzes the mechanisms, applications, and potential of encapsulation technologies in ruminant production. Special emphasis is placed on how these delivery systems represent a significant advance toward precision nutrition. Indeed, the efficacy of encapsulation for microbiota manipulation and toxicological challenges for safe and sustainable implementation is discussed. This critical review addresses the following questions: (1) Under what conditions could encapsulation offer real advantages over traditional additives in ruminant livestock? (2) Are there biologically significant differences between nano- and microencapsulation in the ruminal environment? (3) How are changes in the ruminal microbiota translated into productive performance and environmental impact? (4) What is the balance between productive and environmental sustainability benefits versus the toxicological risks of nanomaterials?

## 1 Introduction

### 1.1 Global challenges and sustainability in livestock production

The impact of food production on climate change is a global concern affecting livestock systems, particularly in developing regions where population growth and improved quality of life increase food demand (1). In the livestock sector, improving feed efficiency is crucial as it reduces environmental impact and increases profitability for producers by decreasing manure and methane gas production (2). However, it is important to recognize the fundamental role of ruminants in the ecology of different ecosystems by transforming low-quality forage resources such as cellulose into high-nutritional-value foods like meat and milk (3). Globally, livestock contributes about 14.5% of the greenhouse gas emissions produced by human activity, with enteric methane being the most significant, representing 40% of livestock emissions (4). Moreover, methane production represents an energy loss of approximately 12% in animals (5). In recent decades, encapsulation technology has developed notably, proving to be an essential tool in diverse industries such as food, pharmaceutical, and nutraceutical. This process involves protecting active substances by coating them with different and immiscible materials, forming capsules that preserve and control the release of these compounds (6). Therefore, exploring new technologies for including bioactive substances with anti-methanogenic effects is important to develop more ethical, sustainable, and environmentally responsible livestock production focused on greenhouse gas mitigation (7).

### 1.2 The ruminal and intestinal microbiome as a therapeutic target in ruminants

Ruminant animals have evolved to utilize difficult-to-digest cellulosic foods thanks to microorganisms inhabiting an anaerobic and methanogenic chamber called the rumen (8). This feature allows ruminants to efficiently convert complex polysaccharides such as cellulose and hemicellulose, indigestible for humans, into high-nutritional-value foods. This digestive capacity results from the proper functioning of microbial communities in the ruminal cavity (9). Manipulating the microbiota of this chamber through diet composition and additive use can influence animal health and production by altering fermentation (3).

The ruminal microbiota composition includes bacteria, protozoa, archaea, and fungi with varying abundance and diversity (10). These ruminal microorganisms are anaerobic, and several produce methane gas through fermentation (3). Factors such as abundance and diversity allow coordinated and complex metabolic cascades, including various cross-feeding networks generating trophic levels among ruminal microorganisms (11).

Recently, the mammalian intestine has also been recognized as an important microbial ecosystem site (12). In ruminants, the intestinal microbiome functions as a dynamic ecosystem characterized by metabolic activity dynamics, changes in microbial communities, and production of fermented acids that significantly regulate host nutrition and immune response (3). Genomic studies have shown that *Bacteroidetes* and *Firmicutes* members predominate in the rumen and intestine (3).

For years, indiscriminate use of additives such as antibiotics in livestock has generated a serious problem: bacterial resistance, which gained more relevance after the COVID-19 pandemic. This is a main reason why the search for equally effective but safer natural alternatives has gained importance, such as plant extracts containing bioactive compounds like saponins, tannins, polyphenols, and flavonoids that can modulate ruminal fermentation and reduce pollutant gasses from livestock production. This strategy of using plant secondary compounds is highly relevant due to their antimicrobial, antioxidant, and anti-inflammatory activities, among others, such as reducing methane emissions (13). However, most of these secondary compounds tend to be susceptible to oxidative degradation, generating free radicals that compromise their bioactivity (14).

### 1.3 Encapsulation: a precision nutrition strategy to overcome bioactive compound instability

The use of traditional additives to optimize the quality of animal feed often faces limitations in terms of effectiveness, cost- benefit, and environmental impact. Therefore, biotechnology has established itself as an innovative alternative in animal nutrition, with nanotechnology standing out as an emerging discipline that enables the manipulation of materials at the atomic and molecular scale (15, 16).

Protecting high-value bioactive compounds such as micronutrients, followed by controlled release in the gastrointestinal system, can be achieved through encapsulation techniques (17). Encapsulation is a process where a core or encapsulated material in our case, probiotics and/or bioactive compounds, is surrounded or protected by a wall material, which may consist of one or multiple layers, resulting in micro- or nanometric-sized structures (18). The goal is to protect and enhance bioactive compound functionality to increase bioavailability (19). Besides protecting bioactives from degradation caused by environmental conditions, it potentiates their bioactivity (18).

One main use of nanoscale delivery systems is achieving controlled release of their content under specific conditions, such as certain pH or temperature values (20). Depending on the objective, they can overcome biological barriers and prevent modifications of bioactive compounds (21).

These delivery systems have led to ruminant nutrition for better nutrient use, reducing food rejection due to unpleasant flavors and increasing animal performance. They facilitate the protection of essential volatile nutrients like vitamins, minerals, probiotics, and additives from degradation in the rumen (22). Additionally, in ruminant-derived products, these practices have contributed to improved product quality by enhancing flavor, texture, and shelf life, masking unpalatable compounds, releasing flavor enhancers, and reducing oxidation in meat and dairy products. However, implementation still faces challenges related to costs, formulation complexities, and regulatory approval (22).

#### 1.3.1 Microencapsulated probiotics

The modulation of ruminal microbiota through probiotic supplementation has gained attention as a strategy to improve health and productive performance in ruminants (23). However, the efficiency of conventional freeze-dried probiotics is often limited by their susceptibility to degradation under the harsh conditions of the gastrointestinal tract, which prevents them from reaching their sites of action in a fully functional manner. Microencapsulation has emerged as a technological solution to this limitation, offering physical protection that improves the stability, bioavailability, and ruminal functionality of probiotics (24).

Despite the consensus on the benefits of encapsulation, the magnitude and nature of the responses vary considerably between studies, suggesting that efficacy is not universal but depends on multiple factors. Ramon Estevez et al. (24) reported that microencapsulated probiotics improved fiber digestibility and dry matter intake in ruminants, with *in vivo* trials involving 270 animals showing an increase in daily weight gain of 115 g/animal compared to sodium monensin. However, it remains to be determined whether this response is reproducible across different production systems or groups of different breeds. Yadav et al. (25) demonstrated that encapsulated formulations of *Limosilactobacillus reuteri* SW23 improved intestinal health indicators and growth parameters in calves more effectively than non-encapsulated forms, highlighting that protection during storage and digestive transit is critical for probiotic viability at the site of action. Nevertheless, the choice of encapsulation method, microencapsulation by spray drying versus lyophilization, appears to influence the results, although direct comparisons between these techniques under identical conditions are scarce.

Mechanistically, the positive effects of encapsulation may be linked to increased bacterial survival and colonization. El-Sayed et al. (26) showed that *Enterococcus faecium* encapsulated with sodium alginate by extrusion survived acidic pH but required protection against bile salts, suggesting that single-layer encapsulation may be insufficient for complete gastrointestinal protection. The microencapsulated form shifted ruminal fermentation toward increased production of short-chain fatty acids and high-value protein, probably due to stimulation of fibrolytic activity. Similarly, Chang et al. (27) used electrospinning to encapsulate *Lactobacillus acidophilus*, achieving prolonged stability in ruminal and intestinal fluids for 48 h, while non-encapsulated bacteria showed rapid decline.

Notably, the relative abundance of *L. acidophilus* increased in the rumen of supplemented groups, indicating that encapsulation not only protects but also favors colonization. However, whether such colonization translates into sustained functional benefits beyond the immediate supplementation periods requires further research.

Together, these findings indicate that microencapsulation improves the efficiency of probiotics, but the variability in responses among the strains studied, the encapsulation technique, and experimental conditions underscores the need for comparative and systematic studies to identify optimal combinations for specific production contexts.

#### 1.3.2 Encapsulated phytochemicals (essential oils and bioactive compounds)

Plant-derived bioactive compounds, including polyphenols, flavonoids, and essential oils, have been extensively studied for their potential to modulate ruminal fermentation, improve animal health, and reduce methane emissions (28, 29). However, their practical application in ruminant nutrition is limited precisely by inherent physicochemical limitations: many are volatile, sensitive to environmental factors, and susceptible to rapid degradation by ruminal microbiota, which compromises their bioavailability and efficacy before reaching the sites of action (30, 31).

The fate of these compounds in the digestive tract of ruminants remains poorly understood. While in monogastrics their absorption and metabolism are relatively well characterized, studies in ruminants reveal a more complex picture. Berger et al. (32) observed early plasma peaks of quercetin following intraruminal administration, suggesting partial absorption directly from the rumen, despite retention times exceeding 1 h. Similarly, Lundh et al. (33) reported a rapid appearance of isoflavones in the plasma of sheep fed estrogenic silage, further supporting the possibility of ruminal absorption. Flavonoids that escape ruminal degradation can be absorbed in the small intestine, as demonstrated by Gohlke et al. (34) after intraduodenal infusion of quercetin. These observations highlight that the site and extent of absorption are variable and depend on the physicochemical properties of the compound and the digestive dynamics of the host.

It is important to highlight that not all bioactive compounds generate positive responses. The effects are highly dose-dependent, and exceeding optimal dietary levels can be counterproductive, leading to reduced feed intake, altered fermentation, or toxicity (35). This narrow therapeutic window underscores the need for more precise delivery systems. Encapsulation offers a strategy to protect these compounds during gastrointestinal transit, modulate their release, and improve their bioavailability while minimizing adverse effects (35).

Essential oil (EO) blends have demonstrated synergistic effects superior to those observed when used individually. These EO combinations have been proposed as alternatives to conventional antibiotics, with the potential to reduce bacterial resistance, improve digestion, and optimize final product quality (36, 37). Some of these products are already commercially available, and Table 1 shows some of the reported effects of some of these blends, such as Phyto Rumen Plus®, whose composition is divided into three equal parts: one-third corresponds to a total phytogenic blend including microencapsulated cinnamon and oregano EO in a 50:50 ratio; another third contains non-encapsulated cinnamon and oregano EO in the same proportion; and the final third consists of turmeric extract (with 5% curcumin) and tannic acid (38).

**Table 1:** Encapsulated bioactive compounds evaluated in ruminants and related models: encapsulation techniques and main findings.

Bioactive compound	Encapsulating material	Encapsulation technique/others	Stability/efficacy	Results	References
Methanolic extract *Moringa oleifera*	Chitosan	Ionotropic gelation	Not reported	Ruminal fermentation and digestibility improved, methane reduced with *M. oleifera* extract, but more studies recommended to optimize dose and adaptability	(113)
Lemongrass powder, mangosteen peel powder	Chitosan	Spray-drying	Not reported	At 6% concentration and forage:concentrate ratio 60:40, improved ruminal fermentation, increased protein synthesis, reduced methanogens and CH4	(114)
*Yucca schidigera* extract, chitosan	Chitosan	Ionotropic gelation	Not reported	Nanoextract of *Y. schidigera* reduced hydrogen sulfide production and improved dry matter digestibility. Nanochitosan reduced methane production by 49% at 48 h	(63)
*Eucalyptus citriodora* essential oil	Chitosan	Emulsification (232 nm)	Not reported	Nanoencapsulated essential oil showed greater efficacy than free extract in inhibiting hatching and larval development of *Haemonchus contortus*	(44)
*Curcuma longa*	Poly(methyl ethyl acrylate)	Polymer interfacial deposition method	≥90%	Supplementation of lambs with curcumin nanocapsules improved growth, antioxidant, and anti-inflammatory responses	(115)
Acacia tannin extract	Maltodextrin and gum Arabic—potato starch	Spray drying	Encapsulation efficiency decreases with increasing core material (Acacia Tannin Extract) concentration; higher for maltodextrin-gum than starch	Methane reduction was marginal due to low loading percentages; other wall materials or encapsulation methods recommended to improve results	(116)
Mimosa tannin extract (*Acacia mearnsii*)	Sunflower oil	S/O/W dispersion	Not reported	Mimosa tannin, free or encapsulated in sunflower oil (20 g/kg DM), modulated ruminal fermentation and reduced methane without affecting intake or gain; encapsulated showed greater methane reduction in sheep	(117)
Thymol, carvacrol, and cinnamaldehyde combined with curcumin and a tannin	Not reported	Phyto Rumen Plus®	Not reported	Phytogenic mix improved gain and final weight; did not affect the 10 predominant rumen bacterial genera	(38)
Carvacrol, thymol, and cinnamaldehyde	Not reported	Enterosan®, Konkreta, Brazil	Not reported	Phytogenic supplementation increased weight gain and reduced leukocytes, indicating a probable anti-inflammatory effect from carvacrol and thymol	(39)
Carvacrol, thymol, cinnamaldehyde combined with curcumin (62% purity)	Not reported	Enterosan®	Not reported	Microencapsulated phytogenics maintain calf performance; curcumin 100 mg/day does not improve production parameters but maintains health by modulating inflammation and immunity	(118)
β-glucosidase	Alginate-sucrose-maltodextrin-chitosan-pectin	Ionotropic gelation	Very good stability after 14 weeks storage at 4 °C. Microbeads with sucrose and maltodextrin: EE = 100% ± 2.16. Microbeads with sucrose: EE = 95.5%. Chitosan microcapsules: EE = 49%. Control alginate microbeads: EE = 3%. Alginate-pectin microbeads: EE = 40.5%	Modification with chitosan, sucrose, and maltodextrin improved β-glucosidase encapsulation (100%), but enzymatic recovery in ruminal fermentation was limited	(119)

Another commercial product containing a blend of microencapsulated essential oils is Enterosan®, which provides 21.55 mg of carvacrol/g, 18.76 mg of thymol/g, and 27.62 mg of cinnamaldehyde/g (36, 37, 39). However, their volatility (e.g., limonene, thymol, carvacrol) and sensitivity to oxygen, light, and enzymatic degradation limit their efficiency in free form (40, 41). Furthermore, concerns about their handling, such as respiratory irritation from vapors, add practical limitations to their use (42). Encapsulation addresses these limitations by providing a physical barrier that can control their release, in addition to protecting the active principles from premature damage and allowing targeted administration to specific places in the gastrointestinal tract.

Despite these advantages, the literature shows considerable heterogeneity in the results. Discrepancies between studies may arise from differences in the composition of the bioactive compound, the encapsulation matrix, particle size, and experimental models (*in vivo* vs. *in vitro*). For example, while some studies report a consistent reduction of methane with encapsulated essential oils (43), Others show variable or insignificant effects depending on the doses and diet composition (44). This variability underscores the need for a systematic evaluation of encapsulation parameters and their interaction with ruminal fermentation dynamics to optimize efficacy in diverse production systems.

#### 1.3.3 Microencapsulated fruit extracts

Microencapsulated fruit byproducts represent a sustainable alternative for modulating ruminal fermentation. Jalal et al. (45) evaluated microencapsulated mango and avocado peel and seed extracts. Some extracts increased digestibility and fermentative efficiency; notably, avocado peel extract reduced methane by 16% and improved acetate:propionate ratio. Microencapsulation preserves antioxidant and antimicrobial activity, facilitating ruminal microbiota modulation and nitrogen use efficiency.

Treatments with mango peel and avocado peel extract resulted in decreased acetate proportion and acetate-to-propionate ratios, indicating a shift in fermentation pathways. Increased propionate in peel extracts of both fruits may result from the increased abundance of *Prevotellaceae* and *Veillonellaceae* families in encapsulated extracts. Results obtained by Ahmed et al. (46) revealed that the use of a garlic extract and citrus extract mixture succeeded in modifying the ruminal microbiome. An increase in propionate-producing families such as *Prevotellaceae* and *Veillonellaceae* was found, along with a reduction in hydrogen-producing bacteria. Regarding archaea, the sequences were primarily classified into two predominant families: *Methanobacteriaceae* and *Methanomassiliicoccaceae*. These families have also been identified as dominant in other studies using the 16S rRNA gene as a reference (47, 48). The application of the garlic and citrus extract mixture produced notable changes in the archaeal community, evidenced by a reduction in the relative abundance of the *Methanobacteiaceae* family, which includes the genus *Methanobrevibacter*, recognized for its key role in methane (CH_4_) production in the rumen.

On the other hand, an increase in the abundance of the *Methanomassiliicoccaceae* family was observed in the groups treated with this mixture, showing a dose-dependent behavior. Thus, while the *Methanobacteriaceae* population significantly decreased compared to the control group, the *Methanomassiliicoccaceae* family significantly increased.

These findings indicate that the mixture modified the composition of the archaeal community by reducing the presence of the family most associated with methane generation and favoring another family that may play a different role in ruminal fermentation.

## 2 Nanotechnology applied to encapsulation: improving the bioavailability and targeted release of bioactive compounds in nutrition

Despite significant advances in the development of encapsulation systems for bioactive compounds, the specialized literature recognizes that there is no universally applicable method. The suitability of a given technique intrinsically depends on the physicochemical properties of the compound to be encapsulated (core), the nature of the encapsulating material, and the functional objective pursued. Therefore, the selection of an encapsulation technique must be based on a thorough analysis of criteria such as molecular weight, polarity, and solubility of the active agent, as well as the morphology, desired particle size, and encapsulation efficiency that can be achieved with each combination of materials and methods (49). In this context, the choice of the encapsulation system size turns out to be a determining factor in its functionality. Nanostructures, compared to their micrometric-sized counterparts, offer a significantly greater surface-to-volume ratio. This morphological characteristic gives nanocapsules a superior capacity to modulate properties such as the apparent solubility of the encapsulated compound, which directly affects its release profile and, ultimately, its bioavailability (50, 51). Reducing the size to the nanoscale can improve solubility, but it also optimizes the pharmacokinetics of bioactive compounds. Nanocapsules allow for more sustained release over time and facilitate precise targeting of the bioactive to specific tissues or organs. This targeting ability is crucial to maximize therapeutic or nutraceutical efficacy, potentially minimizing required doses and off-target effects (52, 53). In the field of ruminant nutrition, these advantages open the door to the design of programmed-release additives that can help modulate ruminal and intestinal microbiota, increasing benefits for the host.

### 2.1 Principles of encapsulation: protection of bioactive compounds in the gastrointestinal tract

The incorporation of functional components, such as bioactive compounds, prebiotics, and probiotics, into the feed matrix aims to improve the nutritional value of feeds, as well as animal health and productivity (46). These substances, derived from plant, marine, animal, and microbial sources, have well-documented potential to address challenges in animal health due to their antioxidant, anti-inflammatory, and antimicrobial properties (54, 55). However, a critical obstacle for their practical application in ruminants is their inherent instability. Factors such as light, oxygen, interactions with the feed matrix, and, fundamentally, the extreme pH conditions and enzymatic and microbial activity throughout the gastrointestinal tract (GIT) can degrade these compounds before they reach their desired site of action (56).

Here is where encapsulation emerges as a fundamental technological tool to ensure the efficacy of these bioactives. Defined as a process that traps an active substance (core) within a carrier material (matrix or coating), encapsulation creates particles ranging from a few nanometers to several micrometers (57, 58). The main objective is to create a physical barrier that protects the compound of interest, thus overcoming the limitations mentioned (56).

The benefits of this approach are multiple and include:

(a) Protection of the bioactive against premature degradation in the rumen.
(b) Masking of unpleasant flavors that could reduce feed palatability.
(c) Prevention of undesirable interactions with other diet components.
(d) Modulation of release, allowing controlled administration targeted to specific segments of the GIT, such as the small intestine, to maximize absorption and bioavailability (30, 56).

### 2.2 Controlled release mechanisms and their relevance in ruminal systems

The key to realizing the benefits of encapsulation lies in controlled release (CR) systems. Unlike immediate release, CR systems are designed to release bioactives at predetermined rates and/or locations, independently of local environmental conditions, thus minimizing ingredient loss before absorption or function (31, 59).

The physicochemical mechanisms governing release from capsules are diverse and may occur simultaneously or sequentially. The most relevant in animal nutrition include:

(a) Diffusion of the compound through the matrix or capsule pores.
(b) Enzymatic or hydrolytic degradation of the encapsulating material (erosion).
(c) Fragmentation or dissolution of the matrix.
(d) Swelling of the polymer forming the capsule, increasing its permeability (60).

Understanding these mechanisms and how to control them is essential to design the encapsulation system that best protects and releases its content at an appropriate site, such as specific portions of the GIT, aiming to bypass to the abomasum and intestine or modify the ruminal microbiota. The administration of bioactive compounds in ruminant nutrition, although promising, faces the challenge of instability in the complex rumen ecosystem. A limitation for free extracts lies in the hydrolytic efficiency of the ruminal microbiota. *In vitro* studies with *Yucca schidigera* extract, whose potential may be compromised by rumen degradation, show that although initially slow, degradation accelerates drastically between 6 and 8 h after incubation starts (61). Other studies present a more challenging scenario: direct rumen infusion of *Costus speciosus* rhizome extract, rich in saponins, reports much faster hydrolysis, completed within the first hour of ruminal exposure (62). While studies with free extracts report almost complete degradation within the first hours of incubation (61, 62), work with protected formulations reveals substantially different fermentative dynamics, especially at later stages. This persistence of biological activity manifests in several parameters at 48 h. One of the most notable findings is the ability of the nanoencapsulated extract to sustainably mitigate harmful trace gasses. In carbohydrate-based diet fermentations, the nanoencapsulated version reduced carbon monoxide (CO) production by 67.7% and hydrogen sulfide (H_2_S) by 30.3% at the end of the 48-h cycle, compared to the control group (63). This efficacy was not limited to a single diet type; in substrates with 18% protein, addition of 1 mL of the nanoencapsulated extract also decreased CO by 64.7% (64). These data suggest that the encapsulation matrix protects the active compounds, allowing them to modulate microbial metabolic pathways responsible for producing these gasses over a prolonged period. In contrast, the impact on methane (CH_4_) production was more complex and diet-dependent. Results indicate a non-uniform response: while in high-carbohydrate diets most doses increased methane production, a specific dose of 1 mL achieved a 15.3% reduction at 48 h in a high-carbohydrate diet (63). Conversely, in forage-based fermentative systems, such as corn silage and oat hay, the effect was consistently suppressive, decreasing asymptotic methane production (65). This dual effect underscores the importance of considering the interaction between additive, dose, and base diet composition when evaluating the antimethanogenic potential of this technology. Beyond gas profiles, ruminal fermentation evaluation revealed positive effects on microbial ecosystem productivity. Inclusion of the nanoencapsulated extract was associated with increased short-chain fatty acid (SCFA) concentration and metabolizable energy (ME) values across various substrates, including different forage types and protein levels (63, 66). Under specific experimental conditions, this ME increase reached up to 25.3% compared to the control (63). It is noteworthy that these improvements in fermentative efficiency occurred without negatively affecting dry matter digestibility (DMD), which remained at optimal levels for microbial function (63). Together, these findings indicate that prolonged release of bioactives not only maintains their functionality over time but may also translate into improved efficiency as the ruminal microbiota degrades and ferments feed. In summary, contrasting results obtained with nanoencapsulated extract against literature on free extracts (61, 62) allows affirming that encapsulation technology fundamentally modifies the kinetic action of *Yucca schidigera*. The presence of significant effects at 48 h, both in mitigating pollutant gasses and improving energy parameters, confirms that the prolonged release system protects active principles from premature hydrolysis, ensuring their bioavailability and functionality throughout the entire fermentative cycle (63).

### 2.3 Matrix materials and encapsulation techniques used in ruminant additive formulation

The choice of technique and coating material is crucial to ensure active compound survival and controlled release. Various approaches have been examined, classified into microencapsulation (spray drying, freeze-drying, extrusion, emulsion systems) and nanoencapsulation (nanoemulsions, solid lipid nanoparticles, nanohydrogels, and electrospinning, Table 2) (67).

**Table 2:** Encapsulation technologies for bioactive compounds in animal nutrition: materials, particle sizes, efficiency, and targeted release.

Technique	Common materials	Average size	Encapsulation efficiency	Target/release site	Advantages	Limitations	References
Spray drying	Maltodextrin, gums (arabic, mesquite)	1–100 μm	High for liposoluble compounds	Variable, more frequently in the rumen	Low cost and scalable to large volumes	Some compounds to encapsulate are very heat-sensitive	(68, 70)
Extrusion	Alginate	500–2,000 μm	Very high	Intestine, ruminal bypass	Economical	Low production speed and large particle sizes	(72, 73)
Nanoemulsions	Lipids, surfactants	20–200 nm	Very good for lipophilic	Can improve overall bioavailability	Very good stability	High equipment cost	(76, 77)
Ionic gelation	Chitosan, alginate	<1 μm	Medium-High	Intestinal	Size control	Difficult to scale to large volumes	(75, 76)
SLN (solid lipid nanoparticles)	Solid lipids	50–999 nm	High	Controlled release in intestine	Biocompatible, good protection of bioactives	Reports of expulsion during storage	(82, 83)

#### 2.3.1 Spray drying

Spray drying is a widely used and economical technique for encapsulating bioactive compounds and nutrients. During atomization and dehydration, thermal and shear stresses can cause some degradation. To counter this, bioactives are stabilized with carrier materials, highlighting proteins and peptides for their ability to reduce surface tension and form protective films around particles (68). This method effectively encapsulates heat-sensitive compounds (69). Microencapsulated products usually have a matrix-type structure, with sizes ranging from a few microns to several tens. Efficacy is higher for nonpolar, low-volatility, and low molecular weight compounds (69). Particles are typically spherical without cracks or fissures (70).

#### 2.3.2 Freeze-drying

Freeze-drying is a highly valued drying technique, producing high-quality products with microporous structures, facilitating excellent rehydration capacity (71). The process involves freezing the product and subsequently removing water by ice sublimation. Common encapsulating agents include gum arabic, starches, whey protein, and maltodextrin (Table 2). Unlike spray drying, freeze-dried particles generally exhibit a glassy, broken, or spongy structure due to sublimation (70). Its main disadvantage is considerable energy consumption.

#### 2.3.3 Extrusion

Extrusion is a physical-mechanical encapsulation technique producing particles sized between 50 and 2,000 μm. It is economical, scalable to large production, and does not require organic solvents, high temperatures, or extreme pH conditions (72). Hot or cold extrusion reduces water activity in foods. However, it may struggle with high-viscosity solutions, and encapsulation efficiency is usually lower than that of spray drying (72). Disadvantages relate to large particle formation and low production speed, which can be compensated for by modifications such as electrostatic extrusion or multiple nozzles (73).

#### 2.3.4 Ionotropic gelation

Ionotropic gelation (IG) is a method of creating micro- or nanoparticles by exploiting electrostatic contact between oppositely charged ions, resulting in phase separation and polymer-rich gel formation (74). IG is a specific technique producing micro- and nanoparticles through electrostatic interaction between two ions, provided one component is a polymer (75). The bioactive compound is trapped within polymer chains. Despite simplicity, the approach is hindered by limited particle size control, costly equipment requirements, and complexities in scaling production (76).

#### 2.3.5 Nanoemulsions

Nanoemulsions are advanced colloidal systems formed by microdroplets sized nanometrically, generally between 20 and 200 nm, giving them remarkable physical stability (77). These systems are ideal for encapsulating, protecting, and controlled releasing lipophilic bioactive compounds such as essential oils and antioxidants. Their unique properties, such as high surface area and optical transparency, help overcome stability and functionality issues of bioactives under different environmental conditions (77). Nanoemulsions are produced by high-energy (ultrasonic, high-speed homogenizers) and low-energy methods (78).

#### 2.3.6 Nanofibers

Nanofibers, long and thin structures made of food-grade biopolymers, are an effective platform for encapsulating and controlling the release of hydrophobic compounds. For example, electrospraying has been used to encapsulate DHA in zein nanofibers, improving oxidation stability (79). Electrospinning is a key fabrication technique applying an electrostatic field to a polymer solution (80). Resulting nanofibers feature a high surface-to-volume ratio and controllable porosity, making them useful for transporting and releasing sensitive bioactives (81).

#### 2.3.7 Solid lipid nanoparticles

Nano-structured lipid carrier systems have been extensively studied. Among them, solid lipid nanoparticles (SLN) stand out as compound delivery nanosystems made of biocompatible and biodegradable lipids in a crystalline state (82). Initially prepared as nanoemulsions, they are then solidified to form a nano-stable structure. SLNs offer encapsulation that improves stability and bioavailability of bioactive compounds due to their consistent nanometric size and solid lipid matrix, reducing bioactive loss (83).

### 2.4 Effects of encapsulated additives on productivity and host efficiency

Most nanotechnology products designed for *in vivo* applications via the oral route consist of nanostructures primarily composed of organic compounds such as proteins, polysaccharides, phospholipids, and lipids. In contrast, nanostructures composed mostly of inorganic materials like metals or metal oxides (e.g., gold, silver, iron oxide, titanium oxide) are developed for medical imaging diagnostics, combined therapies, food safety; an example of which are biosensors developed for detecting pathogenic agents in food and protective nanomaterials for intelligent packaging that extend the shelf life of food (84).

Throughout their coevolution, the digestive system microbiota and host have established a mutually beneficial relationship. The microbiota contributes to nutrient extraction from ingested food, while the host provides a warm, nourished environment essential for maintaining a relatively stable intestinal ecosystem. When this microbial community is imbalanced, a phenomenon known as dysbiosis occurs (85). Moreover, even if ingested nanoparticles are not absorbed, they may cause toxic effects and alter the normal composition of the host microbiota (86). Changes in microbiota balance can also influence nanomaterial absorption. For example, lipopolysaccharides (LPS), molecules produced by all Gram-negative bacteria in the colon, offer additional adhesion points for nanoparticles. This interaction may enhance nanomedicine absorption by prolonging particle retention time in the intestine (87).

Currently, the livestock industry explores various strategies to optimize the quality of animal products such as meat, milk, and eggs. An emerging trend is “biofortification,” supplementing animal feed with encapsulated bioactive compounds to improve absorption and efficacy (88).

Routine antibiotic use in animal production has caused serious problems, including antibiotic-resistant bacteria, affecting livestock health, and potentially leaving residues in meat products (89). In this context, condensed tannins have shown positive effects by inhibiting ruminal biohydrogenation (90), improving meat and milk nutritional quality, especially by increasing conjugated linoleic acid (CLA) (91). For this action to be effective, encapsulated tannins must be released in the rumen, where biohydrogenation is modulated (56).

## 3 Nanotechnology and sustainability in livestock production

The diversity of encapsulation techniques and materials available has driven a growing number of studies aimed at evaluating their potential in ruminant nutrition. However, methodological heterogeneity, both in the techniques employed and in the experimental models used, makes direct comparison between studies and the identification of consistent patterns difficult. Table 1 synthesizes some studies conducted in cattle and related models (chickens, sheep, and *in vitro* models), in order to contrast methodological approaches and evaluate the consistency of the findings reported across different experimental systems, and to analyze their potential impact on livestock production and environmental challenges.

### 3.1 Greenhouse gas mitigation (methane and ammonia)

The literature on bioactive compounds to reduce ruminal methanogenesis is ambiguous and reports variable results (92). This variability is illustrated by studies showing contradictory effects: while a combination of encapsulated phytobiotics reduced protozoa and gas emissions in lambs (93), the same mixture increased methane emissions in steers, possibly due to nonspecific antibacterial effects (94). Likewise, low doses of encapsulated essential oils showed no significant effect on emissions (95).

**Figure 1:**
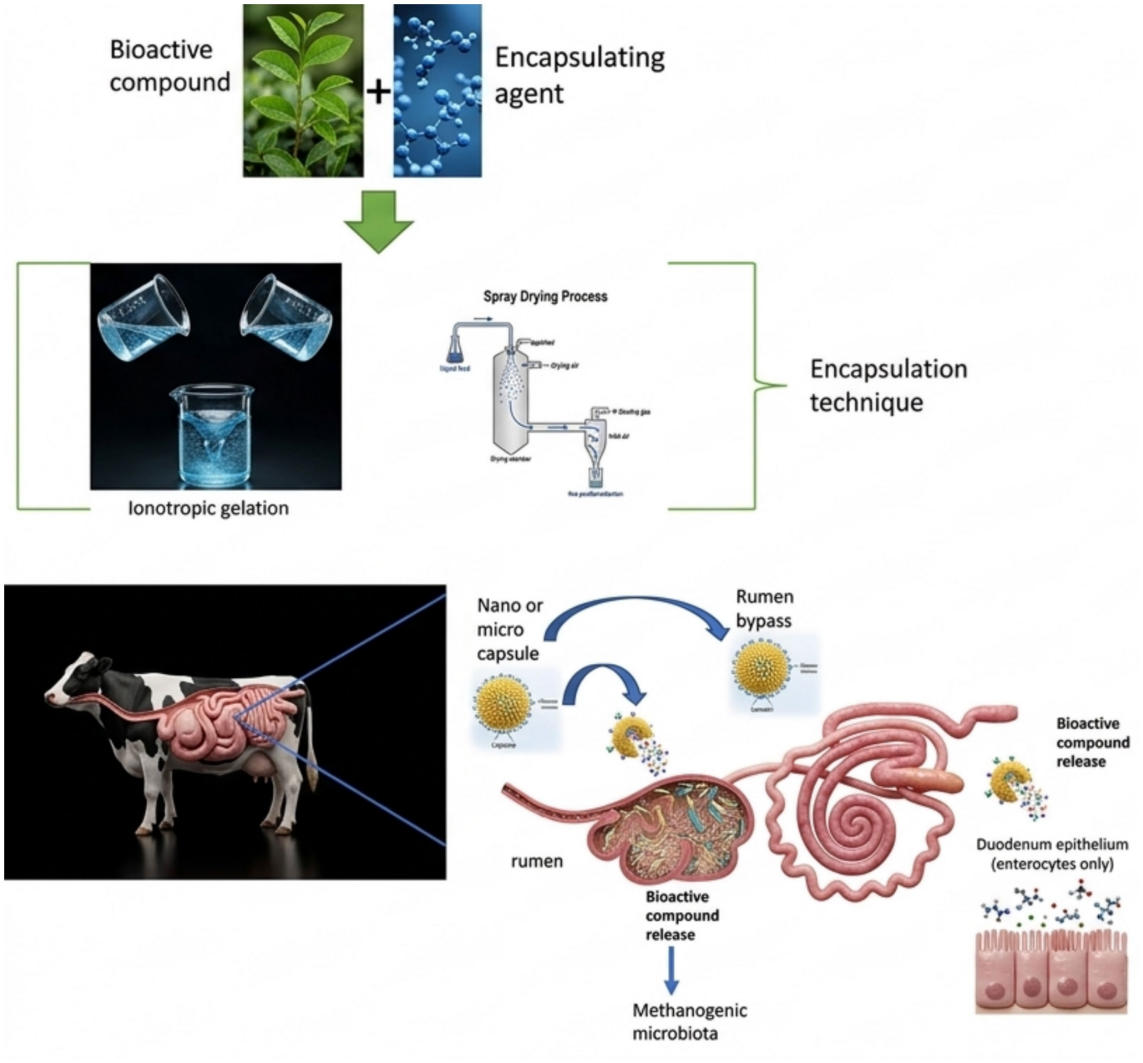
Scheme of the extraction and encapsulation process of plant bioactive compounds and their transit in the ruminant.

Despite inconsistency, potential exists for certain compounds. Some polyphenols have been observed to redirect ruminal fermentation toward higher energy-yielding metabolic routes or selectively inhibit methanogenic archaea, potentially reducing methane synthesis (96). To achieve consistent effects, microencapsulation technology emerges as a promising strategy (Figure 1). For example, microencapsulation of condensed tannins using materials like pork lard or palm oil has shown reduced total gas and methane production *in vitro* (97).

A more recent and specific approach was evaluated by Phupaboon et al. (43), who studied stability and bioaccessibility of microencapsulated plant essential oils. They used formulations such as garlic extract encapsulated with black soldier fly protein (BSF-GOE) and rice bran oil nanoemulsion (RBRBOE). Applying these in 60:40 forage-to-concentrate diets significantly improved *in vitro* ruminal fermentation, increasing digestibility and volatile fatty acid production while considerably reducing methane emission.

The combination of garlic extract encapsulated with black soldier fly protein (BSF-GOE) and rice bran oil nanoemulsion (RBRBOE) showed a selective effect on ruminal microbiota. It did not negatively affect key fermentation bacteria populations such as cellulolytics (*Fibrobacter succinogenes*, *Ruminococcus albus*, *Ruminococcus flavefaciens*), acidogens (*Megasphaera elsdenii*, *Butyrivibrio fibrisolvens*), or those involved in protein synthesis and hydrogenation (*B. fibrisolvens*, *Butyrivibrio proteoclasticus*). Conversely, it significantly reduced specific methanogen abundance (*Methanobacterium methylutens*, *Methanosphaera stadtmanae*, *Methanobrevibacter furmicicum*), decreasing their population to less than 3.5% compared to control after 24 h (43). These findings suggest this mixture may be an effective strategy to mitigate methane emissions without harming beneficial rumen bacteria.

On the other hand, the plant *Mitragyna speciosa*, traditionally used in Thailand, contains bioactive compounds with antioxidant properties that positively influence ruminal fermentation and reduce methane production. In an *in vitro* study (98), showed that supplementation with microencapsulated *Mitragyna* leaf extract improved dry matter degradation, increased ammonia nitrogen concentration and volatile fatty acid production, especially propionate, while significantly reducing methane generation. An increase in cellulolytic bacteria and *Butyrivibrio fibrisolvens* populations was also observed, along with a reduction in *Methanobacteriales methanogens.* The following table shows some examples (Table 3).

**Table 3:** Bioactive compounds and their impact on ruminal microbiota: mechanisms of action, consistency of findings, and knowledge gaps.

Bioactive compound	Reported effects on microbiota	Proposed mechanism	Consistency across studies	Knowledge gaps	References
Flavonoids	↑ Abundance and diversity of *Fibrobacter succinogenes* ↓ Populations of *Ruminococcus albus* and *Ruminococcus flavefaciens* ↓ Ciliated protozoa and hydrogenotrophic methanogens	Competition for hydrogen between bacteria and methanogens; direct inhibition of protozoa-associated methanogens	Consistent in *in vitro* studies; limited *in vivo* validation in ruminants	Effects of suboptimal doses and interaction with high-concentrate diets remain unknown	(120, 121)
Tannins	↑ Butyrate-producing bacteria, *Bifidobacterium*, and *Lactobacillus* ↓ Protozoa, methanogens, and *Ruminococcus albus* Suppression of archaeal communities with concurrent increase in total bacterial population	Inhibition of microbial growth through tannin-protein complex formation; modulation of ruminal redox environment	Variable results depending on dose, tannin type (condensed vs. hydrolyzable), and basal diet	Limited information on long-term effects on microbiome stability and microbial adaptation	(105, 106, 122, 123)
Essential oils	↑ Abundance of *Succinivibrio* and *Bacteroides* Shift in fermentation pattern toward propionate at the expense of acetate	Disruption of microbial membrane permeability; selection of tolerant populations	Highly dependent on oil composition and concentration; contradictory *in vivo* vs. *in vitro* findings	Molecular mechanisms poorly understood; potential for microbial resistance development	(108)
Saponins	↓ Protozoal population and associated methanogens	Interaction with sterol groups in protozoal membranes, causing cell lysis	Consistent in protozoa reduction; variable indirect effects on methanogens	Impact on non-target bacterial communities and protozoal adaptation requires further investigation	(91, 124)

In conclusion, the action of bioactive compounds such as oregano oil, nitrates, and *Quillaja saponaria* extracts to modulate microbiota and reduce methane depends on effective delivery. Encapsulation positions itself as a fundamental tool to overcome free formulation disadvantages, allowing controlled release that maximizes anti-methanogenic benefits, protects basic ruminal fermentation, and facilitates practical application in emission mitigation strategies (99).

## 4 Challenges, risks, and future perspectives

Early approaches in nano and microencapsulation techniques in animal nutrition have employed hydrocolloids such as alginate, chitosan, or maltodextrin, among others, as well as waxes and copolymers to protect compounds like urea (100). However, most of these studies are *in vitro*, with high control of environmental conditions and variables, which limits their extrapolation to real production systems. The extreme conditions of the ruminal tract, high microbial load, variable pH, and enzymatic activity remain the main challenge to ensure the integrity of protected nutrients. Research on encapsulation of minerals and amino acids, whose impact could be directly evaluated in milk and meat quality, remains limited (100). Microencapsulation of probiotics constitutes one of the most promising lines. Although these microorganisms can modulate ruminal microbiota and improve fiber digestion (24–26), their survival during ruminal transit, an anaerobic environment with high microbial competition, remains the main bottleneck. Encapsulation in alginate or chitosan has shown promising results, but observed variability suggests that particle size, matrix composition, and bacterial strain decisively influence efficacy (25, 27). Future studies should optimize these systems and validate them under real production conditions (100). Simultaneously, protein encapsulation with applications such as oral vaccines or functional nutrients emerges as a strategic area. Administering bioactive proteins requires systems that avoid premature degradation in the rumen and allow release in specific intestinal segments. Although research in ruminants is incipient, encapsulation at very small sizes offers a tool to develop targeted delivery systems that could transform health and productive efficiency (100). In this context, lipid nanocarriers present a favorable safety profile for *in vivo* biomolecule administration, unlike metallic nanoparticles or carbon nanotubes (101). Given safety concerns associated with viable microorganisms, postbiotics have emerged as a revolutionary alternative. Defined as bioactive substances derived from probiotics, they can confer health benefits thanks to their chemical structure, safety profile, and prolonged shelf life (102). Being inactivated microorganisms or their metabolites, they eliminate risks of bacterial translocation or opportunistic infections while maintaining desired biological effects. Postbiotic nanoparticles represent a new generation of delivery systems. Derived from Gram-positive and Gram-negative bacteria, as well as other microorganisms and their metabolites, they offer advantages such as prolonging food shelf life and developing functional products (102). Integration of nanoencapsulation with postbiotics constitutes a promising frontier for precision nutrition in ruminants, enabling: However, current evidence mainly comes from monogastrics or *in vitro* models, so extrapolation to the ruminal ecosystem requires more studies and evidence on their stability in ruminal fluid, interaction with native microbiota, and long-term impact on health and productive performance (100). Responsible management of these technologies will require a multidisciplinary approach integrating technological innovation, rigorous toxicological evaluation, and more robust regulatory frameworks.

The costs associated with nanoencapsulation represent a significant barrier, particularly for small and medium-sized enterprises, although economies of scale are expected to facilitate their progressive reduction. This economic limitation is explained by the nature of the available synthesis methods: physical techniques require sophisticated equipment, high temperatures, and intensive energy consumption. Chemical methods, in turn, require costly metal salts and potentially toxic solvents (103). In contrast, green nanotechnology approaches, which employ microorganisms, plants, or their byproducts such as proteins and lipids, have gained attention for offering simpler, faster, environmentally friendly, and lower-cost alternatives (104). This methodological diversity suggests that the economic viability of nanoencapsulation will depend not only on production scale but also on the strategic selection of synthesis routes that balance efficiency, safety, and sustainability.

### 4.1 Toxicity and bioaccumulation: risks for animal health, food safety, and the environment

**Figure 2:**
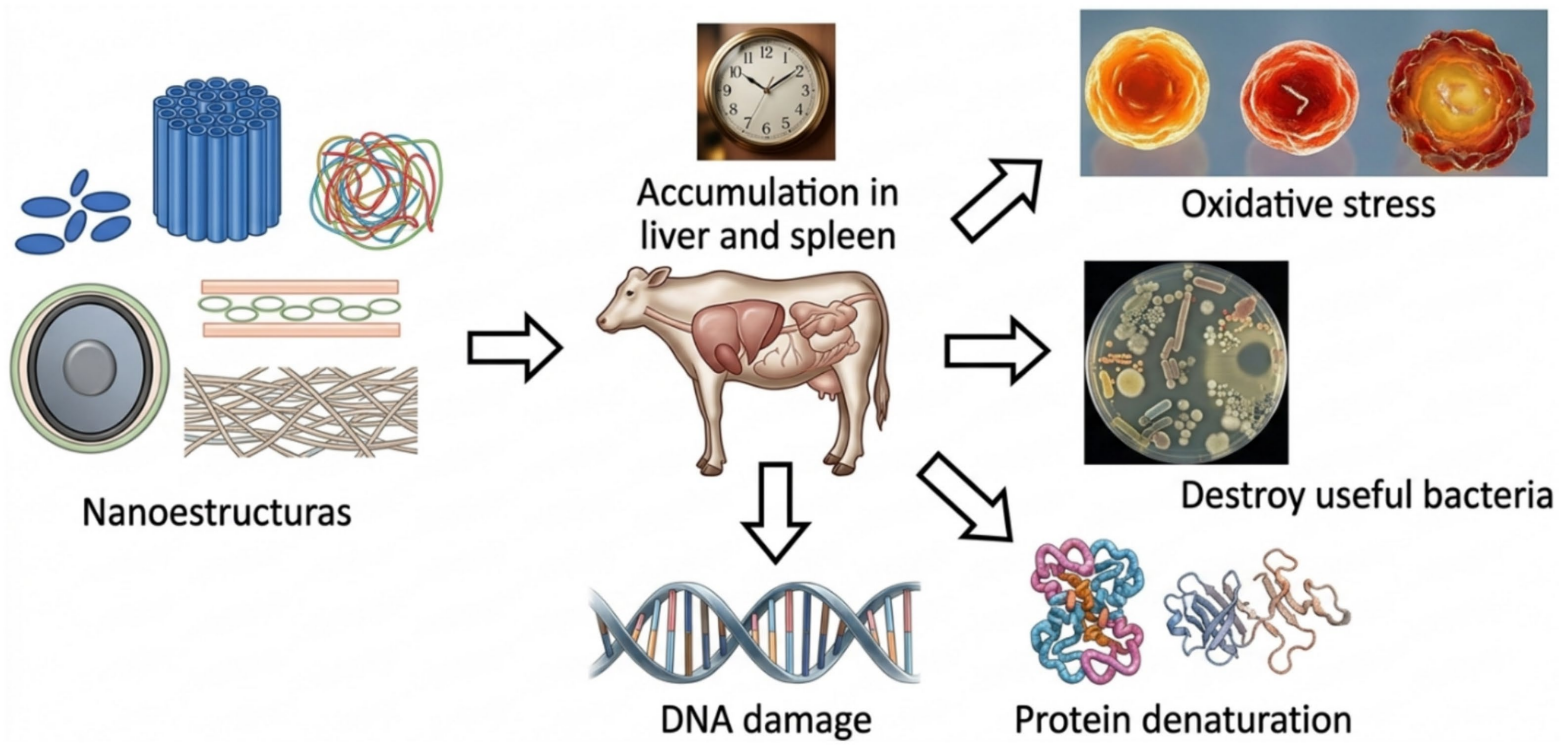
Mechanisms of toxicity induced by nanostructures in ruminants (112).

The incorporation of micro, and nano-structured additives in animal nutrition opens opportunities to improve productive efficiency and the quality of animal-derived foods. However, rigorous evaluation of their possible adverse effects is unavoidable to ensure their safe and sustainable use. Although nanoparticles (NPs) in finished products usually do not come into direct contact with people, animals, or the environment (105), accumulated evidence indicates that certain nanomaterials such as silver, gold, zinc oxide, titanium dioxide, silicon dioxide, and copper oxide can induce toxicity after oral administration (106). This damaging capacity is mainly attributed to their reduced size, which gives them the ability to cross biological barriers, internalize in cells and tissues, potentially triggering adverse physiological responses (107). Prolonged exposure to nanostructures can induce oxidative stress, characterized by overproduction of reactive oxygen species (ROS), leading to damage of proteins, lipids, and nucleic acids (57). This phenomenon is aggravated by the still incomplete knowledge about interaction mechanisms between encapsulating materials and the bioactive compounds they carry. Moreover, continuous accumulation in organs such as kidney, liver, or spleen could negatively alter the composition and functionality of the digestive microbiome, even eradicating microbial populations beneficial to the host (57) (Figure 2). Toxicological profiling of these materials requires standardized methodological approaches since current oral toxicity studies use animal models such as mice, rats, and zebrafish for preclinical data generation. Acute assays, which administer a single high dose of nanoparticles, allow monitoring aspects such as mortality, general well-being, intake patterns, and sensorimotor responses (pupillary reflex, tactile response, acoustic startle) during controlled periods (108). However, the absence of internationally harmonized protocols hinders comparison between studies and extrapolation of results to production species of interest, such as ruminants. Globally, regulatory frameworks for nanomaterials in animal and human food show notably divergent approaches. While regions such as the European Union and Switzerland have incorporated specific provisions for nanomaterials within their food legislation, other countries opt for non-binding guidelines mainly directed at industry (109). This heterogeneity reflects the complexity of managing risks associated with materials whose toxicological properties vary significantly according to their shape, size, agglomeration state, and surface characteristics (110). Regardless of regulatory framework, applicants seeking to market nanotechnology-based products must demonstrate their safe use without posing undue risks to consumers or the environment (109). Nevertheless, adaptation of regulations to the particularities of nanomaterials progresses at different speeds among countries, generating uncertainty for innovation and trade of micro, or nano-structured additives. The ability of nanoparticles to cross biological barriers, including cell membranes and the blood–brain barrier, is a critical factor in their toxicological potential (107). Once internalized, these particles can distribute to various organs and tissues, where they have been associated with a wide variety of pathologies. Several reports link prolonged exposure to nanomaterials such as graphene, iron nanoparticles, silica, silver, carbon nanotubes, titanium dioxide, and gold with neurodegenerative diseases like Parkinson’s, Alzheimer’s, asthma, lung and colon cancer, dermatitis, and cardiac pathologies (111). In the specific context of ruminants, this evidence raises questions about possible long-term effects on animal health, as well as the transfer of nanomaterials to human-consumed products such as milk and meat. Risk assessment must consider both the intrinsic hazard of each nanomaterial and real exposure levels under production conditions, integrating analysis of biodistribution, bioaccumulation, and possible residue presence in edible tissues. Risks associated with nanotechnology are not limited to animal and human health. Residues containing nanomaterials represent a potential source of environmental contamination whose dimensions are not yet fully understood. Available evidence is still insufficient to accurately quantify the amount and behavior of these materials in waste management systems, such as treatment plants or landfills (111). This knowledge gap hinders evaluation of the real environmental impact of nano additives used in animal production and highlights the need for studies addressing the fate, persistence, and ecotoxicity of these materials once released into the environment.

## 5 Conclusion

Encapsulation of additives for ruminant nutrition represents a promising strategy that can overcome the limitations of traditional additives, especially under conditions where the stability and controlled release of bioactive compounds are critical. The protection offered by nano and microencapsulation techniques against adverse environmental and digestive factors helps preserve the functionality of sensitive compounds, such as probiotics, essential oils, and plant extracts, facilitating their effective delivery to the site of action in the rumen. Additionally, encapsulation reduces the perception of unpleasant flavors of these compounds, making them more palatable for the animals. However, the actual effectiveness of these technologies depends on specific variables such as the type of matrix used, dosage, and ruminal system conditions, suggesting that their application must be carefully designed and tailored to particular productive contexts.

Regarding the biological differences between nano and microencapsulation in the ruminal environment, although both techniques offer advantages in protection and targeted release, it remains unclear whether significant differences exist in terms of microbiological and productive impact. Nanoencapsulation may provide greater efficiency in controlled release and bioavailability but also poses greater challenges concerning safety and toxicology, highlighting the need for deeper comparative studies to define its added value over microencapsulation.

Changes induced in the ruminal microbiota by encapsulated additives translate into potential improvements in productive efficiency and mitigation of greenhouse gasses, mainly methane. The modulation of specific microbial communities favors more efficient fermentative pathways and reduces anergy losses. Reflected in parameters such as milk production and reduced pollutant emissions. However, the complexity of the ruminal ecosystem and variability among animals and diets require a multidisciplinary approach to accurately correlate these microbiological changes with consistent productive and environmental outcomes.

In the regulatory field, agencies such as COFEPRIS, the FDA, and EFSA have established guidelines for the use of nanomaterials in food, but the rapid technological evolution often outpaces the adaptive capacity of these frameworks. Risk assessment cannot be limited to physicochemical characterization alone but must include studies on biodistribution, bioaccumulation, and long-term effects in tissues intended for human consumption. A critical knowledge gap persists regarding chronic exposure to low doses of nanoparticles through the food chain, underscoring the need for translational research and international harmonization of regulatory criteria.

Therefore, the balance between productive and environmental benefits versus the potential toxicological risks derived from the use of nanomaterials is a crucial aspect that demands rigorous attention to ensure safe and sustainable implementation. Appropriate regulation and the development of specific protocols for nanomaterial evaluation are essential to mitigate risks, protect animal and human health, and foster social and scientific acceptance of these innovations. In conclusion, nano and microencapsulation constitute valuable tools for ruminant nutrition aimed at sustainability and efficiency, but their optimal application requires greater knowledge of mechanisms of action, safety, and long-term effects. Continued comprehensive research addressing these areas, along with strengthened international regulatory frameworks, is encouraged to maximize the potential of these technologies in real production systems and contribute to more responsible and efficient livestock farming.
